# The Role of Pre-Existing Disturbances in the Effect of Marine Reserves on Coastal Ecosystems: A Modelling Approach

**DOI:** 10.1371/journal.pone.0061207

**Published:** 2013-04-12

**Authors:** Marie Savina, Scott A. Condie, Elizabeth A. Fulton

**Affiliations:** 1 Commonwealth Scientific and Industrial Research Organisation Wealth from Oceans Flagship, Brisbane, Queensland, Australia; 2 Commonwealth Scientific and Industrial Research Organisation Wealth from Oceans Flagship, Hobart, Tasmania, Australia; The Australian National University, Australia

## Abstract

We have used an end-to-end ecosystem model to explore responses over 30 years to coastal no-take reserves covering up to 6% of the fifty thousand square kilometres of continental shelf and slope off the coast of New South Wales (Australia). The model is based on the Atlantis framework, which includes a deterministic, spatially resolved three-dimensional biophysical model that tracks nutrient flows through key biological groups, as well as extraction by a range of fisheries. The model results support previous empirical studies in finding clear benefits of reserves to top predators such as sharks and rays throughout the region, while also showing how many of their major prey groups (including commercial species) experienced significant declines. It was found that the net impact of marine reserves was dependent on the pre-existing levels of disturbance (i.e. fishing pressure), and to a lesser extent on the size of the marine reserves. The high fishing scenario resulted in a strongly perturbed system, where the introduction of marine reserves had clear and mostly direct effects on biomass and functional biodiversity. However, under the lower fishing pressure scenario, the introduction of marine reserves caused both direct positive effects, mainly on shark groups, and indirect negative effects through trophic cascades. Our study illustrates the need to carefully align the design and implementation of marine reserves with policy and management objectives. Trade-offs may exist not only between fisheries and conservation objectives, but also among conservation objectives.

## Introduction

Marine reserves (i.e. no-take areas) are widely used as a tool for marine conservation. Recent reviews demonstrate, on average, positive effects of reserve protection on biomass, numerical density, species richness and size of organisms within their boundaries e.g. [1], [2]. However, these studies also show considerable variability in the responses to marine reserves. Two hypotheses have been suggested to explain this variability in Lester et al [2]. The first is limitations in the experimental design of surveys, particularly the lack of temporal and spatial replication, and the small number of species sampled. The second is sensitivity of the response to conditions around the reserve (i.e. intensity of fishing outside the reserve and infringement into the reserve), and prior to the establishment of the reserve (i.e. historical fishing pressure). It is this last hypothesis that we test here using an end-to-end ecosystem model.

Until recently, modelling of MPA effects has mainly focussed on fisheries outcomes rather than conservation outcomes and primarily focussed on single species models (see review by Gerber et al [3] and White et al [4]). However, non-spatial trophodynamic models (Ecopath with Ecosim or Ecotroph) are increasingly being used for exploring the effects of marine reserves on food webs [5], [6], [7], [8].

There are now a number of quantitative modelling approaches that explicitly represent spatial habitats, animal movements and high-level trophic interactions, all key processes in the response of ecosystems to the implementation of marine reserves (see recent reviews by Fulton [9] and Rose et al [10], and [11], [12], [13]). To our knowledge, only ECOSPACE (the spatially resolved version of ECOPATH with ECOSIM or EwE [14]) has been used for marine reserves applications [15], [16].This type of modelling enables us to explore the effects of marine reserves at a regional scale (as opposed to a comparison of inside/outside reserves) following recent recommendations from a review on marine protected area performances [17]. In the category of spatial trophodynamic models, Atlantis stands out due to its explicit representation of physical and biogeochemical processes, the use of a single biogeochemical framework allowing all trophic levels to be fully coupled without assumptions about bottom-up or top-down control, and its availability-based diet matrix that allows the trophic structure to evolve over time [9], [18], [19] and [20]. Atlantis has already been used to explore the impacts of marine reserves in a fisheries management context in the Gulf of California [21], as well as along the California coast [22].

Protection of biodiversity is amongst the most frequently legislated driver behind the establishment of marine reserves (e.g. [23]). The metric of biodiversity most commonly used in their design and evaluation is taxonomic diversity or species richness [24], and overall, field data tend to suggest a positive effect of marine reserves (e.g. [2] and [25]). However, it has been argued recently that taxonomic diversity only provides an incomplete view of biodiversity as it does not distinguish between species (e.g. [26] and [27]). Functional biodiversity, defined as the functional multiplicity within a community [28], has been defined as a useful additional concept to taxonomic biodiversity, as it correlates with measures of ecosystem functioning [29], [30], resilience to disturbances [31] and regulation in the flux of matter [32]. Functional traits, i.e. the components of an organism's phenotype that influence ecosystem level processes, are generally considered the units defining functional diversity [26]. While taxonomic biodiversity is beyond the scope of end-to-end ecosystem models, functional biodiversity can be explored, as functional traits are generally - and certainly in our case - used to define the model functional groups (e.g. trophic group, size and mobility – [24], [33]). The effect of marine reserves on functional biodiversity has been only recently investigated, and so far the effect seems to be positive [24], [33], [34]. Whether functional biodiversity is correlated (e.g. [24] and [33]), or not correlated to species diversity (e.g. [34]) is not relevant to our study and will not be discussed here.

Here we have used the Atlantis ecosystem model to explore long-term regional changes in response to reserves off the southeast coast of Australia. By considering a range of fisheries and reserve design scenarios we have been able to infer principles that can be applied more broadly to marine reserve design, management and performance assessment.

## Methods

### Model description

**Figure 1:**
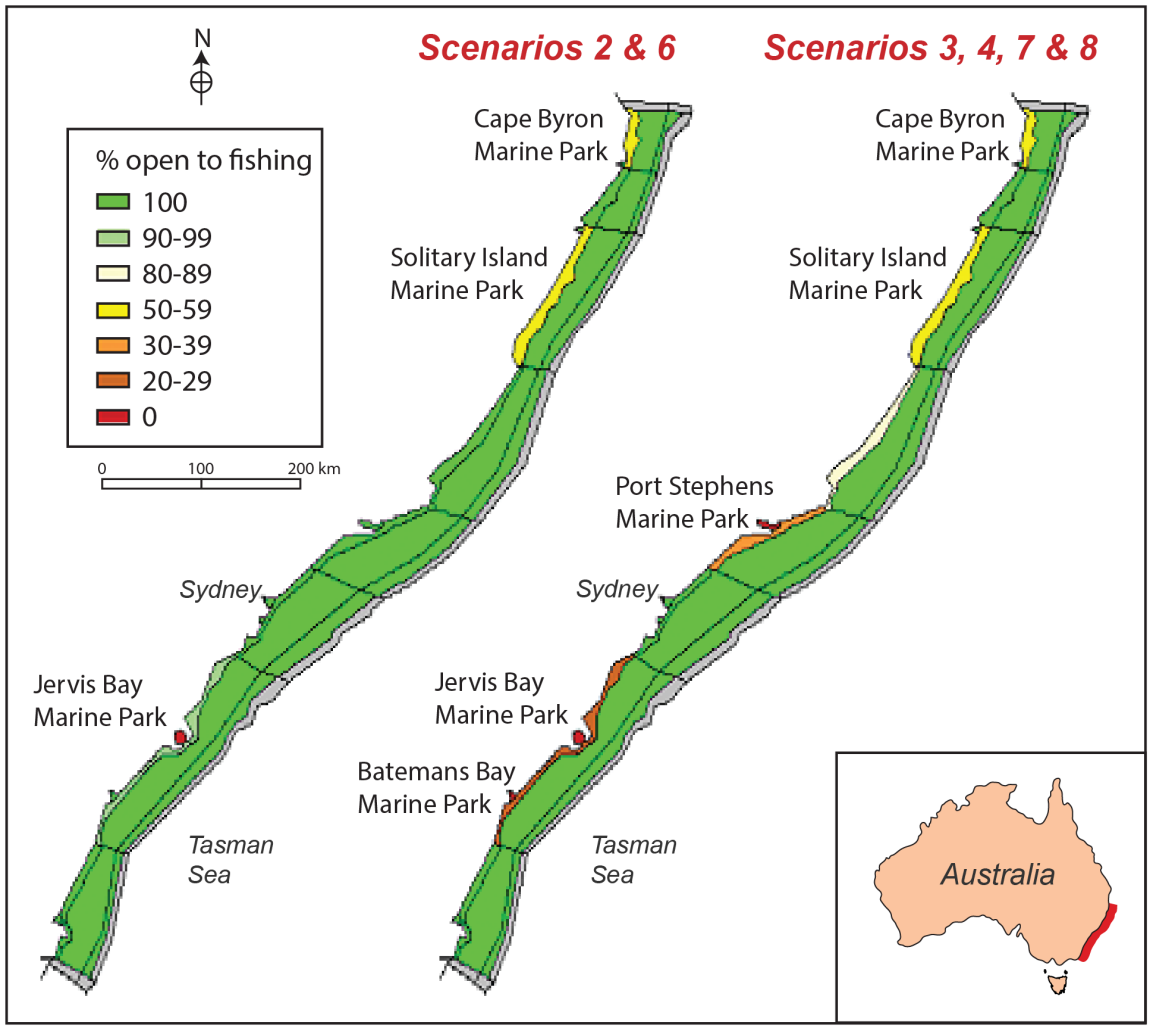
Polygonal cell structure of the model and percentage of model cells open to fishing as a consequence of three marine reserves (left, scenarios 2 and 5) or five marine reserves (right, scenarios 3, and 6). More details in the text.

Atlantis is a nutrient-based biogeochemical model that includes physical forcing, biogeochemical cycling, trophic interactions, and human influences on the ecosystem [9], [18] and [19]. The implementation used here has been described in detail in Savina et al [35]. It covered the length of Australia's New South Wales (NSW) coastline and extended offshore to the upper continental slope (Figure 1). This area was divided into a total of 43 horizontally layered polygonal cells that resolved major bays and estuaries, coastal waters to 50 m, shelf waters to 200 m, and upper slope waters to 800 m. Alongshore divisions were based on land-use characteristics, coastal morphology [36], and the broader pelagic bioregional structure [37]. Vertically, the model resolved a single sediment layer and five water column layers selected on the basis of the general vertical zonation of water properties and pelagic organisms (interfaces at 20, 50, 100 and 200 m).

The model time-step was set at 0.5 days so as to resolve key physical processes (e.g. large-scale current and eddy transports) and key biological processes. Temperatures and salinities within each cell and three-dimensional physical exchanges between cells were estimated at every time-step utilising archived output from a data-assimilating global ocean circulation model referred to as the Bluelink Reanalysis or BRAN [38]. Estuarine inputs were represented as point sources of freshwater and nutrients based on data from local monitoring programs (centralised in the PINEENA database [39]). Offshore nutrient concentrations followed seasonal cycles based on CARS (CSIRO Atlas of Regional Seas) [37]. Solar irradiance at the sea-surface was specified as a function of latitude and the time of year. At the seafloor, a sediment chemistry sub-model calculated nutrient remineralisation and oxygen exchange [40].

The model included 48 biological groups ranging from phytoplankton and bacteria through to sharks and whales (Table 1). Selection of groups was largely determined by the need to capture key ecosystem functional characteristics with the level of aggregation guided by taxonomy, shared predators and preys, size, turnover rate, and habitat use [41], [42]. Experience with implementing and running Atlantis at larger scales in this region [43] assisted with these selections. A significant number of individual species were also represented in the food-web to address identified management issues related to conservation and fishing. For more details please refer to Savina et al [35].

**Table 1:** Functional groups included in the model.

Categories	Sub-categories	Functional groups	Description
Nutrients		Reduced nitrogen (NHx)	Ammonia (NH3), ammonium (NH4)
		Oxidised nitrogen (NOx)	Nitrate (NO3), nitrite (NO2)
		Dissolved silica	
Detritus		Labile detritus	Rapidly decomposing detritus
		Refractory detritus	Slowly decomposing detritus
		Detrital silica	Biogenic silica
		Carrion	
Bacteria		Benthic bacteria	
		Pelagic bacteria	
Plants	Phytoplankton	Large phytoplankton	Diatoms
		Small phytoplankton	
	Phytobenthos	Macroalgae	
		Seagrass	
Invertebrates	Zooplankton	Gelatinous zooplankton	Salps and medusa
		Large zooplankton	Krill, Chaetognaths
		Mesozooplankton	Copepods
		Small zooplankton	Heterotrophic flagellates
	Nekton	Cephalopods	Squids, calamari, and octopus
		Prawns	Eastern king and school prawns
	Zoobenthos	Meiobenthos	
		Benthic carnivores	Polychaetes mainly
		Benthic deposit feeders	Echinoderms, holothurians, bivalves
		Deep filter-feeders	Sponges, corals, crinoids, bivalves
		Shallow filter-feeders	Sponges, corals, crinoids, bivalves
		Commercial filter-feeders	Scallops and oysters
		Benthic grazers	Abalone, gastropods, echinoderms
		Macrozoobenthos	Crustaceans, Asteroids, molluscs
		Commercial macrozoobenthos	Octopus and commercial crabs
		Lobsters	Shovelnosed and rock lobsters
VERTEBRATES	Bony Fish	Demersal shallow herbivorous	Mullets, luderick, garfish, silver drummer
		Demersal shallow territorials	Pipefish, seahorses, gobies, damselfish, diamondfish
		Demersal shallow omnivores	Flounders, gurnards, wrasses, flathead, whiting, bream, snapper, emperors, eels
		Ocean perch	*Helicolenus spp*.
		Whiting	*Sillago spp*.
		Tiger flathead	Platycephalus richardsoni
		Trevallies	*Caranx spp., Pseudocaranx spp*.
		Demersal deep fish	Dories, whiptails, cardinalfish, hapuku
		Morwongs	Cheilodactylus spp., Nemadactylus spp
		Blue Grenadier	Macruronus novaezelandiae
		Pink ling	Genypterus blacodes
		Warehou and trevalla	Seriolella spp., Hyperoglyphe antarctica
		Redfish	Centroberyx affinis
		Gemfish	Rexea solandri
		Pelagic small planktivores	Pilchards, anchovy, scad
		Pelagic large planktivores	Mackerels
		Pelagic shallow piscivores	Bonito, Mulloway, Teraglin, Australian salmon
		Mesopelagic migratory	Myctophids, frostfish, lancetfish
		Mesopelagic non-migratory	Sternophychids, cyclothene (lightfish)
		Oceanic planktivores	Flying fish, sauries, redbait
		Oceanic piscivores	Tunas, swordfish, billfish
	Sharks	Demersal sharks	Gummy, school, wobbegong, sawsharks
		Reef sharks – grey nurse	Carcharias taurus
		Skates and Rays	
		Pelagic sharks	Whalers, blue, mako, white, tiger, hammerhead
		Dogsharks	*Squalus spp*.
		Spiky dogshark	Squalus megalops
	Mammals	Baleen whales	Humpback, southern right, minke, blue
		Dolphins	Bottlenose
		Toothed whales	Orca
		Pinnipeds	Australian and New Zealand fur seals
	Birds	Sea birds	Albatross, shearwaters, gulls, gannets

The biomass of each lower trophic level functional group was computed over time for each of the model cells in nutrient-based units (mg m^−3^ of nitrogen and silica, which are the limiting elements in the system). Higher trophic levels were followed using an age-structured formulation that allowed for changes in reproduction and mortality that may be critical in the response of populations to marine reserves (e.g. [44]).

The functional groups were linked through local trophic interactions and influenced by environmental and habitat conditions in the water column and bottom sediments. Nutrients, planktonic groups and detritus were exchanged between cells on the basis of physical flows estimated from the aforementioned ocean circulation model. The movements of vertebrates were modelled so as to maintain particular distribution patterns, some of which were constant (e.g. demersal shallow herbivorous), while others varied through the year (in case of spawning or other migrations - e.g. Grey nurse shark *Carcharias taurus*). The demersal shallow territorial group was sedentary (i.e. could not move from one box to the others), and a limited set of groups (pelagic large planktivores and shallow piscivores, oceanic piscivores and planktivores, pelagic sharks) had a foraging behaviour that allowed them to move among boxes to optimise their potential growth (calculated on the basis of food resources, existing local fish density and their swimming speed). Other biological processes explicitly represented in the model included consumption and growth, waste production and decomposition, reproduction, habitat dependency, bioturbation and bioirrigation, and predation and other forms of natural mortality. Detailed mathematical formulations for these processes can be found in [40].

Fishing mortality was prescribed in terms of a realised catch based on historical distributions (i.e. extraction continued until the regulated catch quota was reached). The only exceptions were sharks and rays, for which fishing mortality rates were estimated from the literature.

### Scenarios

Between 3 and 5 reserves were considered in our scenarios (Figure 1). They covered 2.4% (1157 km^2^) or 6.2% (2979 km^2^) of the total modelled domain (48000 km^2^). Although they corresponded in location and total area to the real reserves declared off NSW over the past decade (http://www.mpa.nsw.gov.au/), no attempt was made to replicate the complex mosaic of zones with varying degrees of protection. Instead, reserves were idealised as either strictly no-take areas when the reserves covered whole model cells (Figure 1 – red cells with 0% open to fishing); or as areas where fishing mortality (all groups) was reduced in proportion to the ratio of the reserve size to the model cell size (Figure 1 – orange to light green cells). In the rest of the text, we use the term reserve to refer to both of these cases.

The scenarios used the calibrated model and outputs from the start of 1976, see [35], as initial conditions to explore the potential regional impacts of a system of marine reserves over a 30-year period. To isolate the effects of the marine reserves, catches were held constant at either the historical 1976 levels (referred to here as low fishing scenarios) or double the 1976 levels (high fishing scenarios, in line with the levels of increased pressure seen in historical time series [33]). Individual scenarios varied in the number of reserves (3 or 5) and the level of fishing prior to the introduction of reserves (Table 2).

**Table 2:** Summary of model scenarios. Low historical fishing corresponded to 1976 levels off the NSW coast and high fishing to double this level.

Scenario	No. of reserves			Historical fishing	
	0	3	5	Low	High
1	x			x	
2		x		x	
3			x	x	
4	x				x
5		x			x
6			x		x

### Metrics

The results were analysed for the whole model domain using the following metrics related to changes in the higher functional groups (defined here as vertebrates, cephalopods and prawns) over the 30-year simulations. These metrics are different from the ones used in field studies, which usually compare one or several sites within and outside of the marine reserves, but with no indication of what is happening at the scale of the whole region.

- Change in the *biomass of functional groups* expressed as the ratio of the biomass at the end of a given scenario with reserves in place to the biomass at the end of the corresponding baseline scenario with no reserves (i.e. 1 or 4 in Table 2).
- Change in the *fishery catch value* expressed as the ratio of the total catch value at the end of a given scenario to the total catch value at the end of the corresponding baseline scenario. The average prices for each group were calculated from the NSW Department of Primary Industries catch statistics database (1985–1996). While this metric ignores short-term volatility in prices due to factors such as market preferences, international influences and scarcity, it captures any broad trends in catch composition between high and low value species.
- Change in the *functional diversity index* (Q90 defined below) expressed as the ratio of the functional diversity index at the end of a given scenario to the functional diversity index at the end of the corresponding baseline scenario. Q90 is derived from Kempton's diversity index and adapted to measure functional biodiversity [45]. Refer to Savina et al [35] for more details on the use of the index within the model.

## Results

**Figure 2:**
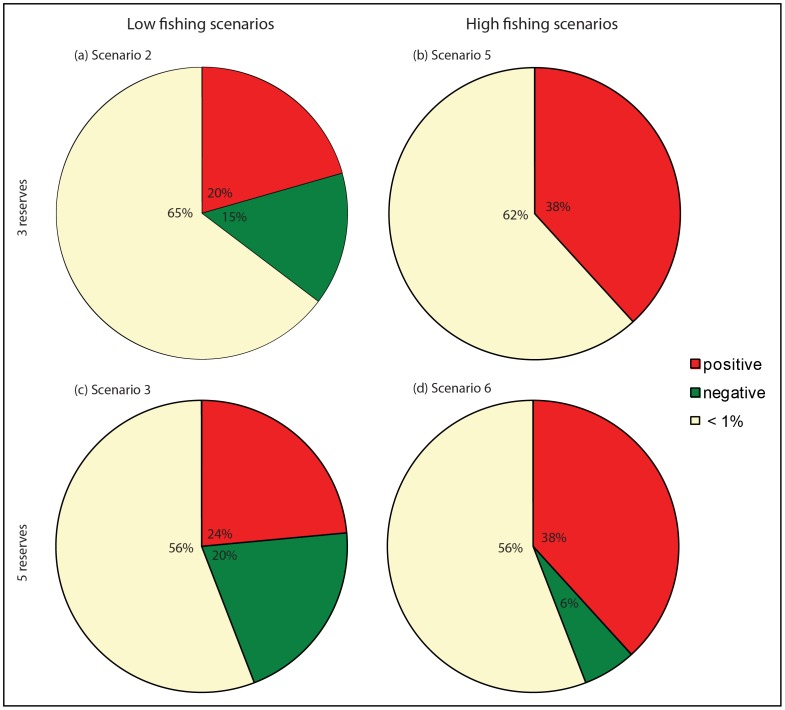
Fraction of all vertebrates, cephalopods and prawns (34 groups) that show an increase, decrease or no significant change in biomass under the four scenarios.

**Figure 3:**
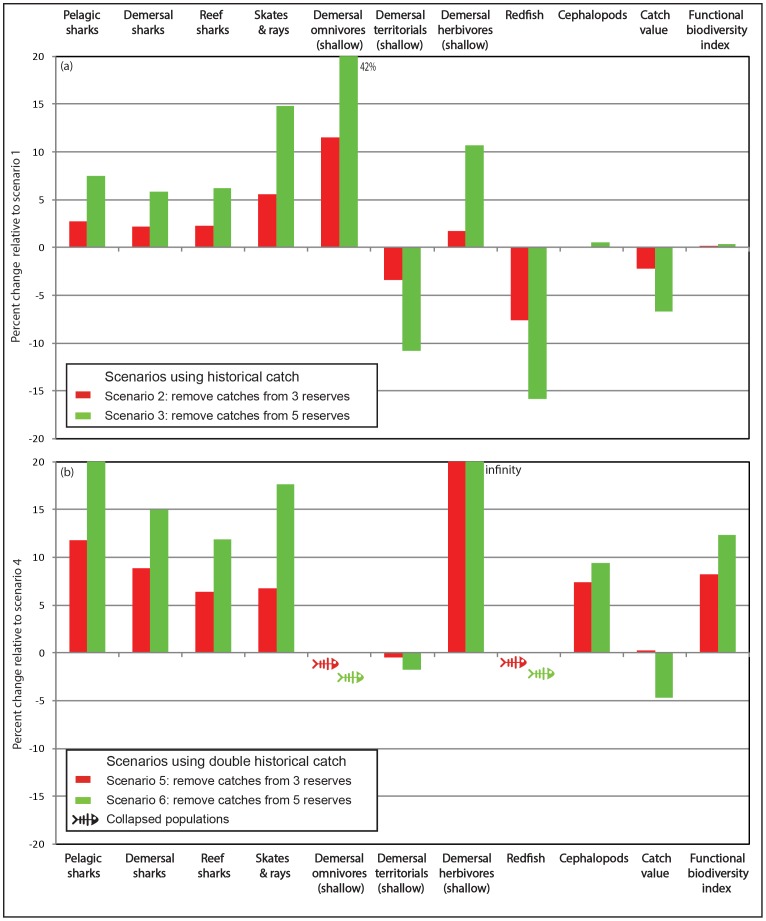
Percentage final biomass changes over 30 years (a) for scenarios 2 and 3 relative to scenario 1 (i.e. low fishing scenarios) (b) for scenarios 5 and 6 relative to scenario 4 (i.e. high fishing scenarios). for the nine functional groups that showed a decrease/increase of more than 8% in at least one of the scenarios. Relative fishery catch value and the biodiversity index are also shown.

The introduction of reserves influenced the biomass of up to 15 of the 34 higher functional groups (i.e. vertebrates, cephalopods and prawns, see Table 1). Under low fishing pressure, 7 of these groups increased in biomass and 5 decreased in biomass in the case of 3 reserves (scenario 2–Figure 2a). The addition of reserves (scenario3 – Figure 2c) caused the total number of groups impacted to increase (15) while the proportion of negatively (7) to positively (8) impacted groups increased slightly. Under high fishing pressure, the proportion of impacted groups stayed roughly the same, but with a much higher proportion of positively impacted groups. In scenario 5, 13 groups increased in biomass and none decreased (Figure 2b). The addition of reserves (scenario 7 – Figure 2d) again caused the total number of groups impacted to increase (15) while increasing slightly the proportion of negatively (2) to positively (13) impacted groups. The strongest responses were amongst groups that lived mostly in the coastal and inner-shelf habitat types protected by the marine reserves (shallow groups in Figure 3a). Under high fishing the presence of reserves prevented the collapse of demersal herbivores, although two other groups (demersal omnivores and redfish *Centroberyx affinis*) collapsed irrespective of their presence (Figure 3b).

When fishing was removed from reserve areas, biomasses increased for pelagic sharks, demersal sharks, reef sharks, skates and rays, demersal omnivores, demersal herbivores, and cephalopods (the last only in the case of the heavy fishing scenarios). In most of these cases the relative changes increased roughly in proportion to the size of the area protected (compare scenarios 2 and 3, or 5 and 6 in Figure 3).

For migratory sharks, such as grey nurse (*Carcharias taurus*), the effects of reduced fishing in reserves necessarily pertained to the entire population. However, even in the case of more sedentary groups, such as demersal omnivores, there was sufficient dispersal in the model for positive effects to extend along most of the coast rather than being restricted to the reserve areas.

The increases in predatory sharks and omnivorous fish tended to have a negative impact on their prey groups. For groups normally being fished in the coastal zone (where the marine reserves are) such as demersal herbivores, increases in predation only partially offset the benefits of reduced fishing pressure. For groups not being fished in the coastal zone, such as demersal territorials (not fished at all) or redfish (*Centroberyx affinis* fished offshore), it resulted in a net decline in scenarios 2 and 3 (Figure 3a). These indirect impacts were evident along the entire NSW coastline, consistent with the widespread increase in predators. Even though redfish only spent their juvenile phase in bays and coastal areas, they were strongly impacted by the increased predation (Figure 3a). They totally collapsed in all the high fishing pressure scenarios (Figure 3b).

Introduction of reserves usually had a small negative impact on fishery catches (<10%, not shown), much of which occurred in estuarine fisheries where the reserve coverage was highest. The associated decline in economic value of catches was smaller still as reductions in total catch were partially offset by increases in the proportion of high value fish groups, such as the shallow demersal omnivores in the scenarios 2 and 3, or the shallow demersal herbivores in the scenarios 5 and 6 (Figure 3). In the scenario 5, the reductions in total catch were indeed completely offset by increases in the proportion of demersal herbivores (Figure 3b).

Removing low fishing from reserves caused a very small increase in the functional diversity index (Figure 3a), while removing high fishing significantly increased the functional diversity index (>10%, Figure 3b). Similar to the biomass results, functional biodiversity increased with the number (and area) of reserves.

## Discussion

In this paper, we used an Atlantis ecosystem model of the NSW continental shelf and upper slope to test the effects of coastal no-take marine reserves at a large regional scale over a 30-year period. The results were analysed using metrics related to the higher functional groups (defined here as vertebrates, cephalopods and prawns) over the 30-year simulations, i.e.: changes in biomass for each functional group and in the functional biodiversity index (Q90), both of which were computed for the whole model domain. This modelling approach contrasts with experimental studies on the impact of marine reserves (which generally measure biomass and biodiversity at a small number of sampling sites inside and outside the marine reserves, and sometimes before and after reserve implementation [2]) and addresses Kemp et al's [17] recommendation that MPA management objectives and monitoring programs should be set at a regional scale so the overall performance can be assessed.

The model results suggest that the ecosystem response to the establishment of marine reserves is dependent upon the pre-existing disturbance state of the system. Here, that disturbance state was expressed as either relatively low fishing pressure (i.e. the 1976 fishing level), or high fishing pressure (i.e. double the 1976 fishing level).

The high fishing scenarios (twice the 1976 fishing level) resulted in a strongly perturbed system, where the introduction of marine reserves had clear and mostly direct effects (i.e. directly through the removal of fishing pressure) on biomass and functional biodiversity. However, under lower 1976 fishing level scenarios, the introduction of marine reserves caused both direct positive effects, mainly on shark groups, and indirect negative effects through trophic cascades (e.g. increased predation on shallow demersal territorials and juvenile redfish *Centroberyx affinis*). Low fishing levels generated an intermediate level of disturbance in the model that sustained relatively high levels of functional biodiversity [35]. In this context, the potential positive effect of reducing fishing pressure on functional biodiversity was offset by the effect of an increased large predator (i.e. sharks) biomass, leading to virtually unchanged Q90 index values. Increased predation has been proposed as an alternative mechanism to competitive exclusion to explain the decline of biodiversity at low levels of disturbance in the intermediate disturbance hypothesis model [46]. Sandin et al [47] similarly observed on coral reefs that with decreasing human disturbance, diversity increased until top predators (including sharks) reached a threshold fraction of the total biomass in the system, leading to declines in their prey.

The ecosystem response also depended on the size of the protected areas, although to a lesser extent than the disturbance rate. All the groups impacted by the establishment of marine reserves show a biomass response proportional to the size/number of the area protected. This is consistent with the meta-analysis findings of Halpern et al [1] and Claudet et al [48], as our metrics measure the system-wide effect (i.e. absolute effect, *sensu* [1]) of marine reserves on the system.

The model results support previous empirical studies e.g. [49]; [50], [51], [52], [53] in finding clear benefits of reserves to top predators, such as sharks and rays, and those benefits extended well beyond the reserve boundaries. Given the identified critical role of top predators in maintaining the structure and function of ecosystems e.g. [54], it can be argued that their conservation should be a major driver and a key performance indicator for the establishment and management of marine reserves.

Our study illustrates the need to carefully align the design and implementation of marine reserves with policy and management objectives. Trade-offs may exist not only between fisheries and conservation objectives, but also among conservation objectives. In a low fishing context, functional biodiversity might be negatively affected by the recovery of large predators, and therefore might not be the best indicator of marine reserve performance. This finding is particularly pertinent given that reserve areas are often selected to minimise conflicts with existing fisheries.

The implications of our model results for the effects of marine reserves on species diversity are uncertain, but interestingly, Hockey and Bosman [55] similarly questioned the use of species richness as an indicator for the management of exploited intertidal rocky shores. The higher species diversity they observed in harvested shores (where there was human collection of intertidal invertebrates) was associated with the dominance of inedible species and small individuals, while undisturbed shores with relatively lower species diversity had a species composition close to or at climax equilibria, which render these communities a far more valuable resource.
